# Pathways between early‐life adversity and adolescent self‐harm: the mediating role of inflammation in the Avon Longitudinal Study of Parents and Children

**DOI:** 10.1111/jcpp.13100

**Published:** 2019-09-04

**Authors:** Abigail Emma Russell, Jon Heron, David Gunnell, Tamsin Ford, Gibran Hemani, Carol Joinson, Paul Moran, Caroline Relton, Matthew Suderman, Becky Mars

**Affiliations:** ^1^ Centre for Academic Mental Health Population Health Sciences University of Bristol Medical School Bristol UK; ^2^ NIHR Biomedical Research Centre University Hospitals Bristol NHS Foundation Trust University of Bristol Bristol UK; ^3^ College of Medicine and Health University of Exeter Exeter UK; ^4^ MRC Integrative Epidemiology Unit University of Bristol Medical School Bristol UK; ^5^ Population Health Sciences University of Bristol Medical School Bristol UK

**Keywords:** Self‐harm, suicide, Avon Longitudinal Study of Parents and Children, adverse childhood experiences, mediation, interleukin‐6, C‐reactive protein, Inflammation

## Abstract

**Background:**

Adverse childhood experiences (ACEs) such as physical and emotional abuse are strongly associated with self‐harm, but mechanisms underlying this relationship are unclear. Inflammation has been linked to both the experience of ACEs and self‐harm or suicide in prior research. This is the first study to examine whether inflammatory markers mediate the association between exposure to ACEs and self‐harm.

**Methods:**

Participants were 4,308 young people from the Avon Longitudinal Study of Parents and Children (ALSPAC), a population‐based birth cohort in the United Kingdom. A structural equation modelling approach was used to fit a mediation model with the number of ACEs experienced between ages 0 and 9 years old (yo), levels of the inflammatory markers interleukin‐6 and C‐reactive protein measured at 9.5 yo, and self‐harm reported at 16 yo.

**Results:**

The mean number of ACEs young people experienced was 1.41 (*SE* 0.03). Higher ACE scores were associated with an increased risk of self‐harm at 16 yo (direct effect relative risk (RR) per additional ACE 1.11, 95% CI 1.05, 1.18, *p* < 0.001). We did not find evidence of an indirect effect of ACEs on self‐harm via inflammation (RR 1.00, 95% CI 1.00, 1.01, *p* = 0.38).

**Conclusions:**

Young people who have been exposed to ACEs are a group at high risk of self‐harm. The association between ACEs and self‐harm does not appear to be mediated by an inflammatory process in childhood, as indexed by peripheral levels of circulating inflammatory markers measured in childhood. Further research is needed to identify alternative psychological and biological mechanisms underlying this relationship.

## Introduction

Exposure to adverse childhood experiences (ACEs) is a well‐established risk factor for self‐harm (Björkenstam, Kosidou, & Björkenstam, 2016; Brown et al., 2018; Cha et al., 2018; Dube et al., 2001; Hughes et al., 2017; Liu, Scopelliti, Pittman, & Zamora, 2018); however, the psychological and biological processes underlying this relationship are unclear. Inflammation has been postulated as a potential candidate mechanism, as a growing number of studies have found an association between inflammatory markers and both ACEs (Baumeister, Akhtar, Ciufolini, Pariante, & Mondelli, 2016; Coelho, Viola, Walss‐Bass, Brietzke, & Grassi‐Oliveira, 2014) and self‐harm or suicide (Black & Miller, 2015). Understanding biological mechanisms between ACEs and self‐harm is important as it offers avenues for treatments that could target these intermediary pathways in order to reduce the risk of self‐harm and suicide.

Findings from a recent systematic review suggest that child maltreatment is associated with elevated levels of the inflammatory markers C‐reactive protein (CRP), interleukin‐6 (IL‐6) and tumour necrosis factor‐alpha (TNF‐α; Baumeister et al., 2016). This supports data from an earlier systematic review that found strong evidence for associations between child maltreatment and CRP, although the authors noted that several studies on IL‐6 did not find these associations (Coelho et al., 2014).

High levels of circulating inflammatory cytokines indicate systemic inflammation, or chronic activation of the immune system. Systemic inflammation is one way through which exposure to ACEs might become biologically embedded, whereby psychological experiences impact on the developmental trajectory of the immune system with resultant effects on health (Berens, Jensen, & Nelson, 2017). The biological inflammatory response in turn triggers a cascade of pathways including affecting levels of serotonin and dopamine, neurotransmitters known to impact on mood regulation and potentially risk of self‐harm (Coelho et al., 2014; Ganguly & Brenhouse, 2015).

Converging lines of evidence from other studies also support an association between inflammation and suicidal behaviour (Batty, Bell, Stamatakis, & Kivimäki, 2016; Black & Miller, 2015). However, most studies are cross‐sectional and are not able to determine the direction of effects between measures (i.e. whether inflammation precedes suicidal behaviour or vice versa). Evidence for the relationship between inflammation and self‐harm in young people is extremely scarce, with a recent review identifying only two studies that have explored this issue (Kim, Szigethy, Melhem, Saghafi, & Brent, 2014). This is an important omission given that adolescence is a peak time for the onset of self‐harm (Hawton, Saunders, & O'Connor, 2012). Existing studies have also predominately been conducted in clinical samples and have focused on suicide attempts that comprise only a small proportion of all self‐harm episodes (Mars et al., 2014).

To our knowledge, this is the first study to explore prospective links between ACEs, inflammation and self‐harm within the same sample. We used data from a population‐based birth cohort to explore whether levels of two key inflammatory markers (IL‐6 and CRP) mediate the association between exposure to ACEs in childhood and self‐harm in adolescence. As early‐life adversity is associated with a range of adverse mental health outcomes (Hughes et al., 2017), we also conducted sensitivity analysis, excluding those with psychiatric disorder, to explore whether any effects are independent of psychopathology.

## Methods

### Sample

Participants were from the Avon Longitudinal Study of Parents and Children (ALSPAC), a population‐based birth cohort in the United Kingdom. ALSPAC initially recruited pregnant women resident in Avon, UK, with expected delivery dates between 1 April 1991 to 31 December 1992 (Boyd et al., 2013; Fraser et al., 2012). The initial number of pregnant women who returned at least one questionnaire or attended a ‘Children in Focus’ clinic by 19/07/99 was 14,541. Of these initial pregnancies, there were a total of 14,676 foetuses, resulting in 14,062 live births and 13,988 children who were alive at 1 year of age. Data were collected via questionnaires and research clinics. The ALSPAC study website contains details of all the data that are available through a fully searchable data dictionary and variable search tool (http://www.bristol.ac.uk/alspac/researchers/our-data/). One of each set of twins and triplets was included in the current sample.

The sample for the current study comprised young people who attended the ‘Focus@9’ clinic at approximately 9.5 years old (yo) and provided a blood sample that was successfully assayed for IL‐6 and CRP. Missing data on exposure, outcome and confounders were imputed using chained equations; thus, our sample was defined by those who had data on the inflammatory mediators. Individuals reporting illness within 7 days prior to the blood test were excluded (*n* = 450) as acute illness impacts on levels of inflammatory markers (Ansar & Ghosh, 2013; Khandaker, Pearson, Zammit, Lewis, & Jones, 2014), giving a total sample of 4,308. Complete data on exposure, mediators and outcome variables were available for 1,619 (the complete case sample; see Figure S1 in the Supporting Information for a flow chart detailing sample definition).

### Ethical considerations

Ethical approval for the study was obtained from the ALSPAC Ethics and Law Committee and the Local Research Ethics Committees.

### Measures

#### Primary outcome: self‐harm

This was defined as an affirmative response to the question ‘Have you ever hurt yourself on purpose in any way (e.g. by taking an overdose of pills, or by cutting yourself)?’ in a questionnaire completed by young people at 16 yo.

#### Exposure: ACEs

Mothers, partners and the study child were asked 288 questions over 27 data collection points about the child's exposure to nine ACEs up to 9 yo. ACEs were child sexual, physical or emotional abuse; parent substance use; parent mental health problems or suicide attempt; violence between parents; parental separation; parental criminal conviction; and child bullying. The first eight are widely used (Hughes et al., 2017). We also included bullying, as it has been used as an ACE in other studies (e.g. Finkelhor, Shattuck, Turner, & Hamby, 2013) and is an important negative life event linked to self‐harm (Hawton et al., 2012). Table S1 contains definitions of each ACE together with the number of contributing questions. ACEs were derived as in Houtepen, Heron, Suderman, Tilling, and Howe (2018), and were considered present if criteria were met at least once by the time the child was 9 yo.

#### Putative mediators

We explored associations with two key inflammatory markers (IL‐6 and CRP), both of which have previously been linked to ACEs and to self‐harm. IL‐6 is a cytokine that has largely pro‐inflammatory effects, and CRP is an acute phase protein produced by the liver in response to IL‐6 and TNF‐α (Tyrka, Parade, Valentine, Eslinger, & Seifer, 2015). Blood samples were spun and frozen immediately after collection at ‐80°C. IL‐6 (pg/L) was measured using high‐sensitivity IL‐6 enzyme‐linked immunosorbent assay (R&D Systems, Abingdon, UK). CRP (mg/L) was measured by automated particle‐enhanced immunoturbidimetric assay (Roche, UK). Interassay coefficients of variation for both outcomes were <5%. These methods have been reported in detail in Khandaker et al. (2014). IL‐6 and CRP were natural‐logarithm‐transformed prior to analysis.

#### Intermediate confounders and covariates

There are four central assumptions relating to confounding in mediation analysis. Exposure–outcome confounding, mediator–outcome confounding and exposure–mediator confounding must be controlled, and no mediator–outcome confounder should be affected by the exposure (VanderWeele, 2016). We treated child internalising and externalising problems and body mass index (BMI) as intermediate confounders. These are factors that may be caused by ACEs and may also causally contribute to both inflammation and self‐harm; that is, they may lie on the causal pathway. SES (maternal education, income and housing tenure), maternal smoking during pregnancy and child sex were treated as confounders of all paths (see Appendix S1 for further methodological details).

BMI was calculated from young people's height and weight at 9.5 yo. The Strengths and Difficulties Questionnaire (SDQ) parent‐report version, a validated and reliable assessment of dimensional child psychopathology (Goodman, 2001), was used to measure internalising and externalising problems when young people were 8 yo. Both subscales were scored out of 20 with higher scores indicating greater problems (Goodman, Lamping, & Ploubidis, 2010).

Smoking during pregnancy was based on maternal self‐report and dichotomised into none or any. Maternal education was reported during pregnancy and categorised as no qualifications, high school qualifications, and advanced‐level or college/university qualification. Maternal report of disposable income was averaged from two questionnaires at 3 and 4 yo. This was equivalised by scaling according to family size, composition and housing benefits and divided into quintiles (Gregg, Propper, & Washbrook, 2007). Housing tenure during pregnancy was dichotomised as rented/housing association versus mortgaged/owned. Child sex was recorded at birth.

### Variables for sensitivity analyses

We conducted a number of additional sensitivity analyses to further explore whether the ACE–self‐harm association was specific to the age at which self‐harm was reported, or whether presence of suicidal intent or psychiatric disorder altered our findings. We examined three secondary outcomes: lifetime history of suicide attempt at 16 yo, multiple episodes of self‐harm in past year at 16 yo, and self‐harm (regardless of suicidal intent) at 21 yo. History of suicide attempt at 16 yo was considered present if young people indicated that they ‘have ever seriously wanted to kill themselves on any occasion where they have hurt themselves’ or the ‘last time [they] hurt themselves it was because [they] wanted to die’. As our primary outcome would also capture those who had self‐harmed only once, we used multiple self‐harm in a sensitivity analysis to represent those with more chronic self‐harm. Young people were asked about the frequency of self‐harm within the past year; we dichotomised this into none or once versus more than once. Self‐harm at 21 yo was measured the same way as the primary outcome. Sensitivity analyses explored whether effects were independent of psychopathology by excluding those with psychiatric disorder, measured using the Development and Well‐Being Assessment (DAWBA) at 15 yo. DSM‐IV diagnosis was generated using information from parents and young people via a computer algorithm (Goodman, Ford, Richards, Gatward, & Meltzer, 2000; Goodman, Heiervang, Collishaw, & Goodman, 2011).

An additional marker of systemic inflammation, the DNA methylation neutrophil‐to‐lymphocyte ratio (mdNLR), was also available for a subsample of 1,000 ALSPAC children (Relton et al., 2015). DNA methylation data and cell type algorithms were used to calculate the ratio (Koestler et al., 2017). During systemic inflammation, neutrophil counts rise while lymphocyte counts drop; higher mdNLR values therefore indicate greater inflammation. mdNLR was derived from DNA methylation profiles as in Ambatipudi et al. (2018). It was natural‐logarithm‐transformed prior to analysis and utilised in a latent variable comprised of mdNLR, IL‐6 and CRP.

### Analysis

**Figure 1:**
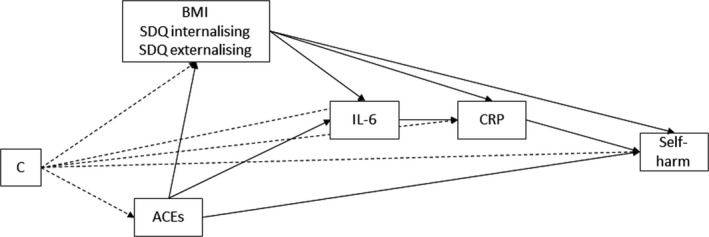
Directed acyclic graph (DAG) of mediation model. Notes: ‘C’ covariates: maternal smoking during pregnancy, child sex, maternal education, income, housing tenure. ACEs, adverse childhood experiences; SDQ, Strengths and Difficulties Questionnaire; BMI, body mass index. Each intermediate confounding path (BMI, SDQs) was specified separately, shown as one for clarity in figure. IL‐6, interleukin‐6; CRP, C‐reactive protein. ACEs were measured from 0 to 9 years of age, IL‐6 and CRP at 9.5 years, and self‐harm at 16 years

We utilised the ACE score as a continuous variable as a likelihood ratio test showed that a categorical model was not a better fit (χ^2^(2) = 2.50, *p* = 0.29). Tetrachoric correlations between ACEs were reported, and Poisson regression of each ACE on the outcome, controlling for covariates, was conducted. A mediation model was fitted to determine whether the association between ACEs from birth to 9 yo and self‐harm at 16 yo was mediated by inflammatory markers at 9.5 yo. As IL‐6 is upstream of CRP in the inflammatory response, both were included in one model (Figure 1). We used a generalised structural equation modelling approach to partition the association between ACE score, IL‐6, CRP and self‐harm into indirect and direct effects with robust standard errors using the gsem and nlcom commands in Stata v15 (StataCorp LLC, College Station, TX) (Gunzler, Chen, Wu, & Zhang, 2013). As self‐harm was not a rare outcome, we used Poisson regression to calculate relative risk (RR; Cummings, 2009). This can be interpreted as the relative risk of self‐harm per additional ACE. Bootstrapping was used to estimate bias‐corrected 95% confidence intervals in complete case data (1,000 replications).

Multiple imputation by chained equations was used to account for missing data (Royston & White, 2011). Our maximum sample had data on both CRP and IL‐6 (*N* = 4308). The study sample was less socioeconomically disadvantaged than the wider cohort, with lower levels of psychopathology (Table S2). ALSPAC has a vast array of auxiliary data that were utilised to make the missing‐at‐random assumption (MAR) that underlies multiple imputation plausible (Sterne et al., 2009). Imputation of each of the study variables used a bespoke combination of auxiliary data including sociodemographic variables, earlier and later measures of variables, measures from a different data collection method/informant, and predictors of each variable. Imputation was performed separately by child sex. Fifty imputed data sets were generated using the *ice* command in Stata v15. The main results reported are based on the imputed data, with complete case results reported in the Supporting Information.

### Sensitivity analyses

We conducted sensitivity analyses with secondary dichotomous outcomes of suicide attempt at 16 yo, multiple self‐harm at 16 yo and self‐harm at 21 yo. We also restricted our model to those without psychiatric disorder at 15 (7.5% excluded). Finally, we excluded those with CRP values of >10 mg/L, as some have argued that values over this threshold are indicative of acute infection/immune activation (Ansar & Ghosh, 2013). A similar threshold or cut‐off has not been proposed or used for IL‐6. We examined whether findings were due to imprecision in measures of inflammatory markers by repeating our mediation analysis with a latent inflammation variable as the mediator, carried out in MPlus version 8 (Muthén & Muthén, Los Angeles, CA) using Monte Carlo integration and unimputed data (*n* = 1,811).

## Results

### Descriptive results (imputed data)

**Figure 2:**
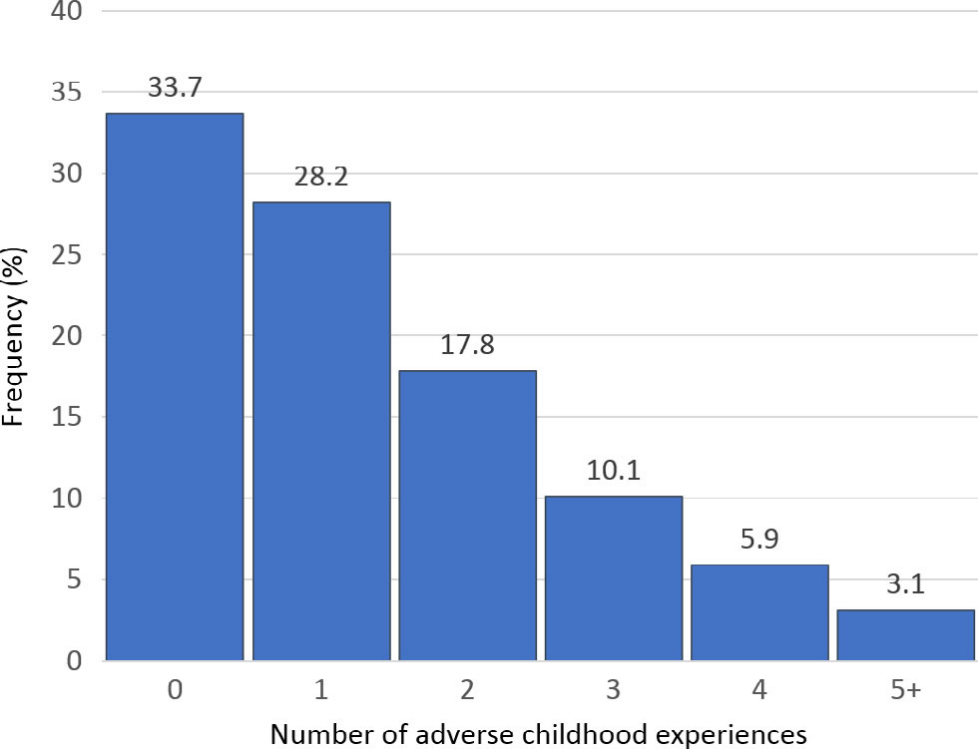
Number of adverse childhood experiences per child (imputed data *N* = 4,308). Notes: 50 imputations. Due to small ns, those experiencing five or more adverse childhood experiences are grouped [Colour figure can be viewed at wileyonlinelibrary.com]

Approximately one quarter of the sample reported self‐harm at 16 yo (24.5%, 95% CI 22.17, 26.83). The mean number of ACEs young people experienced was 1.41 (95% CI 1.35, 1.47). The distribution of ACE scores was positively skewed, with a median of 1 (Figure 2). The frequency of each ACE and their association with self‐harm is shown in Table 1. The most frequently reported ACE was parent mental health problems or suicide attempt (39.3%). Violence between parents (21.7%) and parental separation (21.6%) were the next most common. Sexual abuse was the least experienced, with a frequency of less than 1% (complete case results are shown in Table S3). Of the individual ACEs, the strongest evidence for an association with self‐harm was found for emotional abuse (RR = 1.32, 95% CI 1.07, 1.62) and parental separation (RR = 1.27, 95% CI 1.06, 1.54; Table 1). ACEs were highly correlated with one another, except for sexual abuse and bullying (Table S4). Table 2 shows descriptive statistics for the imputed and complete case samples.

**Table 1:** Exposure to adverse childhood experiences (ACEs) and associations between each ACE and self‐harm at 16 yo (imputed data *N* = 4,308)

Adverse childhood experience	Per cent	95% CI	RR	95% CI	*p*
Sexual abuse	0.8	0.48, 1.19	1.21	0.53, 2.77	0.65
Physical abuse	7.6	6.67, 8.58	1.26	0.96, 1.65	0.09
Emotional abuse	19.1	17.6, 20.5	1.32	1.07, 1.62	0.01
Parent substance use	11.7	10.5, 12.9	1.15	0.88, 1.50	0.30
Parent mental health problems or suicide attempt	39.3	37.7, 41.0	1.17	0.99, 1.38	0.07
Violence between parents	21.7	20.1, 23.4	1.18	0.93, 1.50	0.16
Parental separation	21.6	20.1, 23.0	1.27	1.06, 1.54	0.01
Child experiences bullying	12.7	11.6, 13.8	1.25	0.99, 1.58	0.06
Parent criminal conviction	6.6	5.68, 7.43	1.05	0.74, 1.48	0.80

For definitions of each adverse childhood experience, see Table S1. Fifty imputed data sets. CI, confidence interval; RR, relative risk. Associations between each ACE and self‐harm modelled in Poisson regression controlling for child sex, maternal smoking during pregnancy, income, maternal education and housing tenure.

**Table 2:** Descriptive statistics of study variables, imputed data (*N* = 4,308) and complete case sample (*N* = 1,619)

Variable	Imputed data (*N* = 4,308)		Complete case sample (*N* = 1,619)		
	Mean	*SE*	*n*	Mean	*SD*
ACE score	1.41	1.28	1,619	1.11	1.24
IL‐6 (pg/ml)	1.20	1.45		1.16	1.32
CRP (mg/L)	0.62	1.95		0.57	1.48
BMI age 9	17.6	0.04		17.4	2.55
SDQ internalising problems	2.84	0.04	1,596	2.68	2.58
SDQ externalising problems	4.77	0.06	1,596	4.28	3.19

Variable	%	*SE*	Total n	*n*	%
Self‐harm age 16	24.5	1.19	1,619	304	18.8
Suicide attempt age 16	12.0	1.23	1,619	98	6.1
Multiple self‐harm age 16	16.2	1.31	1,578	159	10.8
Self‐harm age 21	23.0	1.14	1,174	245	20.9
Child sex (female)	48.9	0.76	1,619	891	55.0
Housing tenure (not owned/mortgaged)	17.1	0.58	1,594	150	9.4
Maternal education					
Degree	16.7	0.57	1,608	411	25.6
A‐level	26.9	0.68		500	31.1
GCSE	35.0	0.74		504	31.3
<GCSE	21.5	0.64		193	12.0
Equivalised household income (quintiles)			1,562		
Highest (1)	22.1	0.67		466	29.8
2	21.3	0.66		392	25.1
3	21.5	0.66		323	20.7
4	19.3	0.64		241	15.4
Lowest (5)	15.9	0.60		140	9.0
Maternal smoking during pregnancy (ever)	19.8	0.61	1,619	211	13.0
Psychiatric disorder age 15	7.5	0.56	1,433	59	4.1

SE, standard error; CI, confidence interval; SD, standard deviation; ACE, adverse childhood experiences; IL‐6, interleukin‐6; CRP, C‐reactive protein; BMI, body mass index; SDQ, Strengths and Difficulties Questionnaire (internalising and externalising scores out of 20 with higher scores indicating more problems). Psychiatric disorder age 15 by Development and Well‐Being Assessment (DAWBA) computer algorithm. Fifty imputed data sets were generated.

In regression models exploring associations between the exposure, mediators and outcome, ACE score was associated with both self‐harm and with IL‐6. There was tentative evidence of an association between IL‐6 and self‐harm; however, the confidence interval included the null value. There was no evidence of an association between ACEs and CRP, or CRP and self‐harm (Table S5).

### Mediation results

In the mediation model, higher ACE scores were associated with an increased risk of self‐harm at 16 yo, such that with each additional ACE, an individual was 11% more likely to report self‐harm (direct effect RR 1.11, 95% CI 1.05, 1.17, *p* < 0.001). We did not find evidence of an indirect effect via IL‐6 and CRP, indicating that this association was not mediated by inflammation at 9.5 yo (indirect effect RR 1.00, 95% CI 1.00, 1.01, *p* = 0.38; Table 3, Figure S2). These results were consistent in sensitivity analyses with multiple self‐harm at 16 yo, self‐harm assessed at 21 yo, when excluding those with psychiatric disorder and when excluding those individuals with CRP > 10 mg/L (Table 3). When exploring associations with suicide attempts at 16 yo, the RR was higher than in the main analysis that included both suicidal and nonsuicidal self‐harm (total effect RR 1.22, 95% CI 1.11, 1.33, *p* < 0.001), but this association was not mediated through IL‐6 and CRP. Finally, we treated inflammation as a latent variable, incorporating the mdNLR, and found no evidence of mediation (Table S6). Findings were consistent across the imputed and complete case samples.

**Table 3:** Results of mediation model; association between adverse childhood experiences and self‐harm via inflammatory markers interleukin‐6 and C‐reactive protein (imputed data *N* = 4,308)

Model	Direct effect			Indirect effect via IL‐6 and CRP			Total effect		
	RR	95% CI	*p*	RR	95% CI	*p*	RR	95% CI	*p*
Main analysis									
Self‐harm at age 16	1.11	1.05, 1.17	<0.001	1.00	1.00, 1.00	0.380	1.11	1.05, 1.18	<0.001
Sensitivity analyses									
Self‐harm at age 21	1.12	1.06, 1.18	<0.001	1.00	1.00, 1.01	0.617	1.12	1.06, 1.18	<0.001
Multiple self‐harm at age 16	1.10	1.03, 1.17	0.007	1.00	1.00, 1.00	0.668	1.10	1.03, 1.17	0.007
Suicide attempt at age 16	1.22	1.11, 1.33	<0.001	1.00	1.00, 1.01	0.806	1.22	1.11, 1.33	<0.001
Excluding those with psychiatric disorder at age 15 (*n* = 4,138)	1.11	1.05, 1.17	<0.001	1.00	1.00, 1.01	0.313	1.11	1.05, 1.17	<0.001
Excluding those with CRP values of >10 mg/L excluded (*n* = 4,278)	1.11	1.05, 1.17	<0.001	1.00	1.00, 1.00	0.318	1.11	1.05, 1.18	<0.001

All models adjusted for BMI, internalising and externalising problems as intermediate confounders, and child sex, maternal smoking during pregnancy, income, maternal education and housing tenure as covariates; CRP, C‐reactive protein. Fifty imputed data sets generated. RR, relative risk; CI, confidence interval.

## Discussion

We investigated inflammation as a potential mechanism linking exposure to early‐life adversity and adolescent self‐harm. An association was found between the total number of ACEs and self‐harm, with each additional ACE conferring an eleven per cent increase in risk. The magnitude of these effects is in line with other studies (Dube, Felitti, Dong, Giles, & Anda, 2003) and confirms the established association between adversity and self‐harm found in the literature (Liu et al., 2018). We extend prior work in this area by exploring whether this relationship was mediated by two key inflammatory markers, IL‐6 and CRP; however, we did not find evidence for mediation via inflammation in this sample. We found evidence of a univariate association between ACEs and IL‐6, but not CRP. This lack of association with CRP is in line with findings from a recent meta‐analysis that found no significant association between adversity and CRP, with evidence being weakest for studies in middle childhood (Kuhlman, Horn, Chiang, & Bower, 2019). A growing number of studies have demonstrated the existence of a link between ACEs and inflammation, and between inflammation and self‐harm behaviours (Baumeister et al., 2016; Coelho et al., 2014; Kim et al., 2014; Serafini et al., 2013), highlighting this as a suitable candidate mechanism. However, ours is the first study to provide a direct test of this hypothesis.

There are several plausible explanations for our findings. First, it is possible that the timing of our measures may have had an impact on our ability to detect associations. Much of the literature on ACEs and inflammation focuses on adulthood, with few studies conducted in childhood (Baldwin et al., 2018; Danese et al., 2011; Slopen, Kubzansky, McLaughlin, & Koenen, 2013). The immune system continues to mature throughout adolescence, and it may be that differences in systemic inflammation due to ACEs may not manifest until adulthood (Kuhlman, Chiang, Horn, & Bower, 2017). Our ACE measure was comprehensive, derived from 288 questions, asked between birth and 9 yo; however, there could be sensitive periods during which exposure to ACEs has a greater impact on the developing immune system. Evidence for sensitive periods comes from a study that used slightly different measures of adversity and explored their association with CRP in ALSPAC. ACEs experienced from 6 to 8 yo were associated with CRP; ACEs experienced from 1.5 to 6 yo were not, however, unless included in a cumulative index from 0 to 8 yo (Slopen et al., 2013). Alternatively, associations may differ according to the type of ACE experienced, with more biologically salient ACEs such as physical abuse having stronger effects (Kuhlman et al., 2017). Investigating whether results are impacted by the timing or type of ACE was beyond the scope of the current study, but is an important area for future research.

Research interest in the ACE–inflammation association is increasing. A recent study on victimisation in childhood and adolescence found associations with inflammatory markers by age 18 in females but not males (Baldwin et al., 2018). The construct of victimisation overlaps with several of our ACEs including bullying, exposure to domestic violence, physical and sexual abuse. The relationship between victimisation and inflammation may depend on the type and timing of victimisation. For example, associations between adolescent but not childhood sexual abuse and adult CRP levels have been reported while the same was not found for physical abuse (Bertone‐Johnson, Whitcomb, Missmer, Karlson, & Rich‐Edwards, 2012). Peer victimisation in childhood and adolescence has been associated with higher levels of CRP in midlife (Takizawa, Danese, Maughan, & Arseneault, 2015) and also in young adulthood (Copeland et al., 2014). In addition, peer victimisation within the past week has been found to be associated with an increased inflammatory response to immune challenges in adolescent girls (Giletta et al., 2018). These findings demonstrate that the temporal relationship between ACEs and inflammation is complex, with ACEs having both acute and chronic effects on the immune system. Our measure of ACEs only captured experiences by the age of nine and our measure of inflammatory markers was taken at a much younger age (9.5 years) than in these studies (largely in adulthood), which may explain why we did not find the same association.

Another possibility is that the mechanism through which inflammation impacts on self‐harm does not occur through systemic inflammation, as assessed in this study, but rather as an altered inflammatory response to immune system challenges. Indeed, we did not find evidence that IL‐6 or CRP levels predicted self‐harm. Some previous studies have not found an association between circulating levels of inflammatory markers and ACEs but have instead reported differential IL‐6 responses to both psychological and bacterial challenges (Miller & Chen, 2010).

A third possibility is that prior studies may be detecting inflammatory consequences of self‐harm as opposed to antecedents, as the act of self‐harm itself will trigger an immune response and thus inflammation. Much of the evidence for the inflammation–self‐harm relationship comes from cross‐sectional studies in adult populations, which are unable to establish the direction of association (Brundin, Erhardt, Bryleva, Achtyes, & Postolache, 2015). Our study is also novel in that we explored associations with self‐harm regardless of suicidal intent, whereas prior research has focused on suicidal behaviour. This is important as suicide attempts make up only a small proportion of total self‐harm episodes (Mars et al., 2014). Sensitivity analysis focusing only on suicide attempts found a similar pattern of results to our main findings, yet the total effect was stronger for the suicide attempt outcome than for the main analysis, with the relative risk of self‐harm increasing to 22% per additional ACE.

A further difference between our study and previous research is the use of a population‐based sample. The strongest epidemiological evidence for the inflammation–suicidal behaviour association comes from large studies utilising national registries of healthcare records (Serafini et al., 2013). Cases of self‐harm in these studies will only be recognised when an individual seeks treatment. Those who present to services differ from those who do not, in terms of demographic characteristics, as well as severity and method of self‐harm (Hawton et al., 2012).

A further possible explanation for our findings is that that there is no causal relationship between ACEs and self‐harm operating through inflammation. Indeed, a causal association between ACEs and self‐harm is yet to be empirically established. Two recent studies have demonstrated that part of the association between adversity and self‐harm is attributable to genetic confounding, but also that exposure to adversity confers an additional risk of self‐harm, indicating a small causal influence of adversity on self‐harm (Baldwin et al., 2019; Richmond‐Rakerd et al., 2019). A myriad of social, psychological and biological factors have been proposed to play a role in the aetiology of self‐harm (see Hawton et al., 2012). To rule out the causal role of inflammation, genetically informed methods such as Mendelian randomisation should be used to supplement existing knowledge. If there is no support for a causal association in either direction, further research should focus on other potential causal pathways to elicit potential targets for screening, prevention or intervention.

### Strengths and limitations

Our study is the first to explore the association between ACEs, inflammation and self‐harm. Data were from a large population‐based birth cohort with information on over 4,000 young people. This is important, as most cases of self‐harm are not known to services (Hawton et al., 2012). Our ACEs were prospectively assessed across childhood, and the longitudinal design enabled us to clearly establish the direction of effects between our variables. We also adjusted for a range of confounders and were also able to explore associations with several different measures of inflammation, including IL‐6, CRP and the mdNLR.

Our findings need to be interpreted in the light of several limitations. First, nonresponse and loss to follow‐up have been shown to occur more frequently among individuals with particular characteristics (including socioeconomic disadvantage, ACEs and psychopathology; Wolke et al., 2009) that may lead to bias. In the current study, young people who did not consent to give blood had higher SDQ emotional problems scores than those who provided consent (data available on request). Our study sample was less socioeconomically disadvantaged and experienced less psychopathology than the wider ALSPAC cohort, which may limit the generalisability of our findings. Under the missing‐at‐random assumption, multiple imputation will correct for any biases present in the complete case analyses, and our findings using imputed data were comparable with the complete case. We also conducted a number of sensitivity analyses, and our findings were unchanged, increasing confidence in our conclusions.

Second, ACEs were reported by mothers and fathers/partners (with the exception of bullying) and may have led to an underestimate in prevalence, particularly for ACEs related to abuse. We also did not include neglect as an ACE. Neglect was measured by ALSPAC; however, no suitable questions had been asked by 9 yo, the cut‐off we used to ensure that our measure of ACEs was prior to the measure of inflammatory markers, and thus, we decided not to include it in the current study. Self‐harm was self‐reported by young people at 16 and 21 yo and may be subject to misreporting (Mars et al., 2016). However, estimates of the prevalence in our complete case data (18.8%) are in line with international estimates (Gillies et al., 2018).

In addition, it is possible that ACEs occurring after the age of 9 would increase the risk of self‐harm at 16 yo. However, because of the nature of our mediation model, these effects would not have been captured by the pathway that we measured. This would weaken our ability to detect an indirect effect via inflammation at 9.5 yo. Further studies could control for later ACE exposure or explicitly model this within the analysis.

In conclusion, we found ACEs between the ages of 0 and 9 were associated with an increased risk of adolescent self‐harm, but the association was not mediated by markers of inflammation measured in late childhood. Strategies aimed at preventing ACEs and interventions targeted at those who have been affected may be beneficial for preventing self‐harm behaviours. Future studies are needed to explore the impact of ACE type, and timing of exposure, on self‐harm risk. Further research should also explore alternate biological and psychological pathways through which ACEs might influence risk of self‐harm and suicide. Plausible candidates include HPA axis dysregulation, puberty and epigenetic modifications (Patton et al., 2007; Turecki & Brent, 2016).

**Key points**

- Adverse childhood experiences (ACEs) are a strong predictor of self‐harm; however, the mechanisms underlying this association are unclear. Inflammation has been linked to both ACEs and self‐harm in separate studies.
- In the first study of its kind, we explored whether systemic inflammation, indexed by interleukin‐6 and C‐reactive protein levels, mediated the association between ACEs and adolescent self‐harm.
- We found a strong association between total number of ACEs and self‐harm, with each additional ACE conferring an additional 11% risk of self‐harm at age 16.
- We did not find evidence that this association was mediated by inflammation in childhood, suggesting that inflammatory markers may not be a useful biomarker for self‐harm risk among those exposed to early‐life adversity.

## Supporting information


**Appendix S1.** Confounders in mediation analysis.
**Figure S1.** Participant flow chart.
**Figure S2.** Path diagram of mediation results.
**Table S1.** Adverse childhood experiences (ACEs).
**Table S2.** Descriptive statistics, study sample and ALSPAC core sample.
**Table S3.** Adverse childhood experience frequencies in complete case study sample (*N* = 1619).
**Table S4.** Tetrachoric correlations between adverse childhood experiences (unimputed data, *n* = 2446).
**Table S5.** Univariable and adjusted associations between mediation variables (imputed data *N* = 4308).
**Table S6.** Complete case sensitivity analyses mediation results.
