# Supplementary material for: Pathways between early‐life adversity and adolescent self‐harm: the mediating role of inflammation in the Avon Longitudinal Study of Parents and Children
Source: J Child Psychol Psychiatry. 2019 Sep 4;60(10):1094–103. doi: 10.1111/jcpp.13100 (PMC6771906; doi:10.1111/jcpp.13100)
Supplement: Supplementary file 1 — Appendix S1. Confounders in mediation analysis. Figure S1. Participant flow chart. Figure S2. Path diagram of mediation results. Table S1. Adverse childhood experiences (ACEs). Table S2. Descriptive statistics, study sample and ALSPAC core sample. Table S3. Adverse childhood experience frequencies in complete case study sample (N = 1619). Table S4. Tetrachoric correlations between adverse childhood experiences (unimputed data, n = 2446). Table S5. Univariable and adjusted associations between mediation variables (imputed data N = 4308). Table S6. Complete case sensitivity analyses mediation results. [file JCPP-60-1094-s001.docx]

# **Supporting information – Pathways between early life adversity and adolescent self-harm: the mediating role of inflammation in the Avon Longitudinal Study of Parents and Children (ALSPAC) – by Russell *et al*.**

**Appendix S1.** Confounders in mediation analysis

In-depth discussion of potential confounding variables and their biological and temporal plausibility led us to treat child internalising and externalising problems and body mass index (BMI) as intermediate confounders (i.e. they may lie on the causal pathway); and SES (maternal education, income and housing tenure), maternal smoking during pregnancy and child sex as confounders of all paths.

Confounders in mediation analysis may be situated on the C_XY_, C_XM_ or the C_MY_ paths. In order to determine relevant confounders to include in the mediation analysis, a literature search was conducted to identify factors that are thought to causally influence CRP and IL-6 levels, and self-harm. Risk factors for ACEs were also identified. Confounders on each path had to satisfy the criteria that they could causally influence both variables. Because ACEs could occur any time between birth and age 9, there were limited factors that we considered had potential to causally influence their exposure, so we had several intermediate confounders that could be a result of ACEs, and causally impact on inflammation and on self-harm. Because self-harm was measured six years after CRP/IL-6 levels, careful consideration was given as to which factors may causally influence both inflammation at age 9 and self-harm. The biological plausibility of potential confounders was also considered. This process resulted in the selection of three intermediate confounders that could each putatively by caused by ACEs, and we decided that it was likely that they may causally influence the exposure-mediator (C_XM_) and the exposure-outcome (C_XY_) pathways.

In the case of internalising and externalising problems, we were unable to be wholly confident about the direction of association between these and our exposure and mediating variables. Internalising and externalising problems have been shown to predict higher levels of inflammatory markers (Slopen et al., 2013), and child mental health problems have been shown to be a risk factor for experiencing ACEs: young people who are difficult to parent are vulnerable to ACEs, but it is also likely that experiencing ACEs will lead to child mental health difficulties. Another consideration was that internalising symptoms on the SDQ most proximal to the Focus@9 clinic predicted missing biomarker data. Those who did not give blood were more anxious than those who did, with a mean SDQ emotional problems score of 1.39 (SD 1.67) for those who gave blood; and 1.69 (1.86) for those who did not (independent samples t-test p<0.001).

## **Figure S1.** Participant flow chart

ALSPAC core sample alive at one year N=13988

Multiple births dropped (first born siblings kept in sample) n=13793

CRP and IL-6 data age 9 n=4756

Drop those with current/recent infection: Imputed sample N=4308

Complete case (ACE and self-harm data) n=1619

## **Figure S2.** Path diagram of mediation results

ACEs

Self-harm

CRP

IL-6

direct effect

RR 1.11, 95% CI 1.05, 1.17

Indirect effect

RR 1.00, 95% CI 1.00, 1.01, 1.005, p=0.38

Notes: paths are adjusted for intermediate confounders and covariates: see main manuscript Figure 1. ACEs adverse childhood experiences. IL-6 interleukin-6, CRP c-reactive protein. RR relative risk. CI confidence interval. ACEs were measured from 0-9 years of age, IL-6 and CRP at 9.5 years, and self-harm at 16 years. The indirect effect reported is via both IL-6 and CRP.

## **Table S1.** Adverse Childhood Experiences (ACEs)

| **ACE** | **Definition** | **Number of questions** | **Age range of questions asked** | **Respondents** |
| --- | --- | --- | --- | --- |
| Sexual abuse | Was the child sexually abused | 7 | 18 months to age 9 | Mother |
| Physical abuse | Whether physically cruel to child | 31 | 8 weeks to 9 years | Mother and Partner |
| Emotional abuse | Whether or not mum/partner had been emotionally cruel to the child | 32 | 8 months to 9 years | Mother and Partner |
| Parent substance use | Daily use of cannabis or any use of other drugs. Or, alcohol problem by self-reported problematic use, and saw a doctor because of it | 62 | 8 weeks to 9 years | Mother and Partner |
| Parent mental health problems or suicide attempt | Depression scores (EPDS>12) and medication, presence of schizophrenia, bulimia, anorexia or attempted suicide. | 57 | 8 weeks to 9 years | Mother and Partner |
| Violence between parents | Parent experienced physical cruelty from partner, or displayed (specific types) of violence towards partner | 43 | 8 weeks to 9 years | Mother and Partner |
| Parental separation | Parents divorced or separated. Degree to which this impacted on the child. | 32 | 8 weeks to 9 years | Mother and Partner |
| Bullying | Child bullied | 6 | 8 years-8.5 years | Child |
| Parent convicted | Parent convicted off offence | 18 | 8 weeks to 9 years | Mother and Partner |

Notes: ACE adverse childhood experience. EPDS Edinburgh Postnatal Depression Scale. Several of the ACEs included questions about how much the ACE had impacted on the parent or child.

## **Table S2**. Descriptive statistics, study sample and ALSPAC core sample

| **Description** | Study sample | | ALSPAC core | | p value |
| --- | --- | --- | --- | --- | --- |
|  | (N=4308) | | (N=9,485) | |  |
|  | n | % or mean (SD) | n | % or mean (SD) |  |
| Child sex (female) | 4,308 | 48.89 | 9,485 | 48.18 | 0.443 |
| Housing tenure (not owned/mortgaged) | 4,175 | 16.62 | 8,687 | 31.51 | <0.001 |
| Maternal education | 4,164 |  | 8,092 |  | <0.001 |
| *Degree* |  | 16.95 |  | 10.75 |  |
| *A-level* |  | 26.99 |  | 20.21 |  |
| *GCSE* |  | 35.16 |  | 34.29 |  |
| *<GCSE* |  | 20.89 |  | 34.75 |  |
| Equivalised household income (quintiles) | 3,811 |  | 6,001 |  | <0.001 |
| *Highest (1)* |  | 22.70 |  | 18.78 |  |
| *2* |  | 21.54 |  | 19.00 |  |
| *3* |  | 21.60 |  | 18.81 |  |
| *4* |  | 19.08 |  | 20.28 |  |
| *Lowest (5)* |  | 15.09 |  | 23.13 |  |
| Maternal smoking during pregnancy (yes) | 4,281 | 19.46 | 9,076 | 29.86 | <0.001 |
| Parity | 4,164 |  | 8,597 |  | 0.001 |
| *0* |  | 44.07 |  | 45.12 |  |
| *1* |  | 36.74 |  | 34.17 |  |
| *2* |  | 14.22 |  | 14.31 |  |
| *3+* |  | 4.97 |  | 6.40 |  |
| Birthweight (g) | 4,257 | 3433 (534) | 9,362 | 3388 (557) | <0.001 |
| Maternal age at child birth (years) | 4,308 | 29.1 (4.55) | 9,485 | 27.5 (5.07) | <0.001 |
| White British ethnicity | 4,155 | 98.07 | 8,013 | 97.03 | 0.001 |
| Child psychiatric disorder age 7 | 3,992 | 5.06 | 6,371 | 7.11 | <0.001 |
| Maternal depression | 3,907 | 6.46 (4.56) | 6,420 | 6.94 (4.82) | <0.001 |

Notes: n’s refer to number with relevant data on each variable; ALSPAC core sample is excluding those who died before the age of 1 and second-born twins and the 4,308 individuals who comprise the study sample; p value refers to t-test or χ^2^ of between-group differences; Child psychiatric disorder based on Development and Wellbeing Assessment; Maternal depression measured by the Edinburgh postnatal depression scale, completed by mother 18 weeks into pregnancy

## **Table S3.** Adverse childhood experience frequencies in complete case study sample (N=1619)

| **Adverse childhood experience** | **n** | **Percent** |
| --- | --- | --- |
| Sexual abuse | 5 | 0.31 |
| Physical abuse | 109 | 6.73 |
| Emotional abuse | 247 | 15.26 |
| Parent substance use | 137 | 8.46 |
| Parent mental health problems or suicide | 538 | 33.23 |
| Violence between parents | 284 | 17.54 |
| Parental separation | 210 | 12.97 |
| Child experiences bullying | 180 | 11.12 |
| Parent criminal conviction | 88 | 5.44 |

Notes: For definitions of each adverse childhood experience see Table 1 above. 50 imputed datasets.

## **Table S4.** Tetrachoric correlations between adverse childhood experiences (unimputed data, n=2446)

|  | Sexual abuse | Physical abuse | Emotional abuse | Parent substance use | Parent mental health problems or suicide | Violence between parents | Parental separation | Child experiences bullying |
| --- | --- | --- | --- | --- | --- | --- | --- | --- |
|  |  |  |  |  |  |  |  |  |
| Sexual abuse |  |  |  |  |  |  |  |  |
| Physical abuse | **0.40** |  |  |  |  |  |  |  |
| Emotional abuse | 0.14 | **0.72** |  |  |  |  |  |  |
| Parent substance use | -1.00 | **0.20** | **0.28** |  |  |  |  |  |
| Parent mental health problems or suicide | -0.08 | **0.33** | **0.37** | **0.25** |  |  |  |  |
| Violence between parents | 0.11 | **0.37** | **0.39** | **0.33** | **0.31** |  |  |  |
| Parental separation | **0.33** | **0.22** | **0.30** | **0.16** | **0.28** | **0.33** |  |  |
| Child experiences bullying | -1.00 | 0.02 | 0.04 | 0.03 | 0.05 | 0.07 | 0.05 |  |
| Parent criminal conviction | -1.00 | **0.25** | **0.14** | **0.19** | **0.12** | **0.19** | **0.19** | **0.138** |

Notes: bolded font is significantly correlated at p<0.05

## **Table S5.** Univariable and adjusted associations between mediation variables (imputed data N=4308)

|  | **Unadjusted** | | | **Adjusted** | | |
| --- | --- | --- | --- | --- | --- | --- |
|  |  |  |  |  |  |  |
| **Association between** | **RR** | **95% CI** | **p** | **RR** | **95% CI** | **p** |
| CRP and self-harm | 1.01 | 0.95, 1.09 | 0.684 | 1.01 | 0.94, 1.08 | 0.767 |
| IL-6 and self-harm | 1.08 | 0.99, 1.18 | 0.102 | 1.07 | 0.98, 1.17 | 0.153 |
| ACEs and self-harm | 1.14 | 1.08, 1.21 | <0.001 | 1.11 | 1.05, 1.18 | <0.001 |
|  |  |  |  |  |  |  |
|  | OR | 95% CI | p | OR | 95% CI | p |
| ACEs and IL-6 | 1.03 | 1.01, 1.05 | 0.001 | 1.03 | 1.01, 1.05 | 0.010 |
| ACEs and CRP | 1.00 | 0.98, 1.03 | 0.731 | 1.00 | 0.97, 1.03 | 0.915 |
| IL-6 and CRP | 1.83 | 1.76, 1.89 | <0.001 | 1.82 | 1.76, 1.89 | <0.001 |

Notes: RR relative risk (Poisson regression); OR odds ratio (linear regression); CI confidence interval; CRP c-reactive protein; IL-6 Interleukin-6; ACEs adverse childhood experiences

| **Model** | **Direct effect** | | | **Indirect effect via IL-6 and CRP** | | | **Total effect** | | |
| --- | --- | --- | --- | --- | --- | --- | --- | --- | --- |
|  | **RR** | **95% CI** | **p value** | **RR** | **95% CI** | **p value** | **RR** | **95% CI** | **p value** |
|  |  |  |  |  |  |  |  |  |  |
| Main analysis: outcome self-harm age 16 | 1.19 | 1.10, 1.27 | <0.001 | 1.00 | 0.99, 1.01 | 0.850 | 1.19 | 1.10, 1.27 | <0.001 |
| Sensitivity: outcome self-harm with suicidal intent age 16 | 1.31 | 1.16, 1.51 | <0.001 | 1.00 | 0.99, 1.01 | 0.793 | 1.32 | 1.16, 1.51 | 0.806 |
| Sensitivity: outcome multiple self-harm age 16 (n=1490) | 1.17 | 1.05, 1.29 | 0.003 | 1.00 | 0.99, 1.01 | 0.881 | 1.17 | 1.05, 1.30 | 0.003 |
| Sensitivity: outcome self-harm age 21 | 1.14 | 1.05, 1.23 | <0.001 | 1.00 | 1.00, 1.01 | 0.65 | 1.14 | 1.06, 1.23 | 0.001 |
| Sensitivity: young people without psychiatric disorder at age 15 (n=1293) | 1.18 | 1.08, 1.28 | <0.001 | 1.00 | 0.99, 1.02 | 0.456 | 1.18 | 1.08, 1.28 | <0.001 |
| Sensitivity: CRP values of >10mg/L excluded (n=1610) | 1.19 | 1.10, 1.27 | <0.001 | 1.00 | 0.99, 1.01 | 0.772 | 1.19 | 1.11, 1.27 | <0.001 |
|  |  |  |  | **Indirect effect via latent variable** | | |  |  |  |
| Sensitivity: Latent inflammation variable comprising mdNLR, IL-6 and CRP (n=1811) | 1.18 | 1.10, 1.26 | <0.001 | 1.00 | 0.99, 1.00 | 0.471 | 1.18 | 1.10, 1.25 | <0.001 |

## **Table S6.** Complete case sensitivity analyses mediation results

*Notes: RR relative risk; CI confidence interval; CRP c-reactive protein; IL-6 interleukin 6; CRP c-reactive protein; mdNLR DNA-methylation neutrophil to lymphocyte ratio; bias-corrected confidence intervals after bootstrapping are reported; models are adjusted for BMI, internalising and externalising problems as intermediate confounders, and child sex, maternal smoking during pregnancy, income, maternal education and housing tenure as covariates. n=1619 unless otherwise specified.*
